# Determining Optimal Cutoffs for Exhaled Carbon Monoxide and Salivary Cotinine to Identify Smokers among Korean Americans in a Smoking Cessation Clinical Trial

**DOI:** 10.1155/2021/6678237

**Published:** 2021-02-15

**Authors:** Sun S. Kim, Seongho Kim, Philimon N. Gona

**Affiliations:** ^1^Department of Nursing, University of Massachusetts Boston, USA; ^2^Department of Social Welfare, Korean Bible University, Republic of Korea; ^3^Department of Exercise and Health, University of Massachusetts Boston, USA

## Abstract

**Introduction:**

It is critical to accurately identify individuals who continue to smoke even after treatment, as this may prompt the use of more intensive and effective treatment strategies to help them attain complete abstinence.

**Aims:**

This study examined optimal cutoffs for exhaled carbon monoxide (CO) and salivary cotinine to identify smokers among Korean Americans in a smoking cessation clinical trial.

**Methods:**

CO and cotinine were measured three to four times over 12 months from the quit day. Statistical analysis was conducted using Receiver Operating Characteristic (ROC) curves.

**Results:**

A CO cutoff of 5 parts per million provided robust sensitivity (80.8-98.3%) and perfect specificity (100%), and a salivary cotinine cutoff of level 2 (30-100 ng/ml) provided the best sensitivity (91.2-95.6%) and perfect specificity (100%). Using these cutoffs, the agreement between self-reports and the two biomarkers ranged from 88.6% to 97.7%. The areas under ROC curves (AUCs) of exhaled CO ranged from 0.90 to 0.99, all of which were significant (all *p* values < 0.001), and the AUCs of salivary cotinine ranged from 0.96 to 0.98 (all *p* values < 0.001).

**Conclusion:**

Exhaled CO and salivary cotinine are complementary, and they should be used together to verify smoking abstinence for smokers in a clinical trial.

## 1. Background

The prevalence of current smoking ranged from 19% to 36% among Korean American men and from 4% to 21% among Korean American women [1–3]. Surveys conducted using the English language found lower prevalence than surveys conducted using both Korean and English languages, which was largely attributable to the fact that a majority of Korean American smokers were Korean-speaking male immigrants [4]. Like the substantial decline observed in the general population, smoking prevalence in Korean American men decreased substantially over the past decade [1, 3]. In contrast, a slight upward trend was found among Korean American women.

Although studies are limited, Korean Americans were found to be different in cigarette consumption from other Asian American smokers. For example, Korean Americans were more likely to be daily and moderate to heavy smokers, whereas Chinese and Vietnamese American men were more likely to be nondaily or daily light smokers [5]. Unlike Chinese [6], Japanese [7, 8], and Southeast Asians [9] who had slower nicotine metabolism rates than Whites, Korean Americans exhibited no difference in nicotine metabolism compared to Whites [10]. A smoking topography study reported that daily nicotine intake might not be different between Whites and Korean Americans because the latter seemed to compensate for their lower number of cigarettes per day and low-nicotine-yield cigarettes by smoking with higher puff flows, greater peak puff flows, and much shorter interpuff intervals than the former [11].

Nicotine is the primary addictive agent in tobacco, and P450 2A6 (gene name: CYP2A6) is the primary catalyst of nicotine metabolism [12]. Its blood half-life is approximately 2 hours, and therefore, it is not a useful marker for assessing smoking abstinence [13]. Cotinine is the major proximate metabolite of nicotine and has a relatively longer half-life (i.e., 8-30 hours) than nicotine and allows for the detection of tobacco use even after a few days of abstinence [13]. Accordingly, cotinine is considered the best biomarker for validating smoking cessation except for individuals using nicotine-containing medications or other tobacco products such as electronic cigarettes (e-cigarettes). Although gas chromatography is an accurate quantitative measure of cotinine concentration, the assay is costly. Colorimetric or dipstick immunoassays can measure cotinine in urine and saliva and are relatively inexpensive and straightforward. NicAlert® test kits (Craig Medical Distribution, Inc., Vista, CA, USA) are the most widely used immunoassay and have been compared with alternative methodologies such as gas chromatography [14] and liquid chromatography-tandem mass spectrometry. However, the validity of the immunoassay method is still questionable because of mixed results [14–17].

Another biomarker for smoking is carbon monoxide (CO). Exhaled CO and blood carboxyhemoglobin are highly correlated [18, 19]. Exhaled CO is simple and relatively inexpensive to measure, and there are several types of commercially available breath CO analyzers. The major advantage of CO is that it can detect a current smoker, regardless of whether the individual uses a nicotine-containing medication. However, a critical limitation is the rapid elimination of CO from the body that makes it difficult to identify light smokers [13]. Individuals who smoke only a few cigarettes within 24 hours can be misclassified as nonsmokers. Another limitation is that CO cannot be used to detect the use of noncombustible tobacco products such as smokeless tobacco and e-cigarettes [13].

More than half (53.8%) of a randomly selected sample of Korean American smokers who participated in a nationwide telephone survey reported that they had made a serious quit attempt that lasted at least 24 hours in the year prior [20]. This quit attempt rate is similar to that (55.1%) found in the general population of US adult smokers in 2018 [21]. It is critical to accurately identify individuals who continue to smoke despite treatment such as nicotine replacement therapy (NRT), as this may prompt the use of more intensive and effective treatment strategies to help smokers attain abstinence [22]. The present study was aimed at determining the optimal cutoffs of exhaled CO and salivary cotinine to verify smoking abstinence and examine the sensitivity and specificity of the measures in Korean Americans who participated in a smoking cessation trial.

## 2. Method

This study is a secondary data analysis of a randomized controlled trial of a smoking cessation intervention conducted with Korean Americans. The trial was conducted between 2010 and 2013, with 109 Korean immigrants residing in northeastern regions of the US [23]. Participants were randomized 1 : 1 to either the treatment group or the control group. The treatment group received eight weekly 40-minute counseling sessions of a culturally tailored smoking cessation intervention and NRT. In contrast, the control group received eight weekly 10-minute counseling sessions of a standard smoking cessation intervention and NRT. Primary findings of the randomized controlled trial have been reported elsewhere [23]. In this report, we report findings based on the analysis of 88 participants who completed at least two of four follow-up assessments after the smoking cessation intervention. All participants provided informed consent, and the Institutional Review Board of a university approved the study.

### 2.1. Participants

Individuals were eligible if they (1) self-identified as a Korean descent, (2) were in the age range of 18 years or older, (3) were able to speak and read either Korean or English, (4) had been smoking 10 or more cigarettes daily for the 6 months prior, (5) were willing to use nicotine patches as directed, and (6) were expected to live in the current geographical area for at least one year after enrollment into the study. Exclusion criteria were as follows: (1) any serious mental illnesses (e.g., psychotic disorder), (2) current serious skin disease, (3) current use of any illegal substances, (4) severe alcohol use, or (5) pregnancy or lactation for women.

### 2.2. Measures

Exhaled CO was measured at baseline and 1, 3, 6, and 12 months postquit using the Micro+ Smokerlyzer® (Bedfont Scientific, South Hackensack, NJ, USA). A cutoff of ≥7 parts per million (ppm) was recommended for smoking by the manufacturer. Salivary cotinine concentration was measured using a NicAlert® test kit at 3, 6, and 12 months postquit. Saliva was collected without stimulation, and the test was not done at 1 month postquit because many participants then used nicotine patches provided as part of the smoking cessation intervention. The test kit yields six levels of cotinine concentration from level 0 (0-10 ng/ml) to level 6 (≥2000 ng/ml), and its manufacturer recommends a cutoff of level 1 (10-30 ng/ml) for smoking. We chose to use saliva instead of urine because we could directly observe the collection of saliva samples. We asked participants to spit into a collection tube while in the physical presence of an assessor. We did not perform the saliva test if participants reported using any nicotine-containing medication in the seven days prior.

Self-reported abstinence was assessed at each postquit assessment using the 7-day timeline follow-back of smoking consumption [24]. Therefore, abstinence was determined by a combination of exhaled CO and self-report at 1 month postquit, when the salivary cotinine test was not conducted, and a combination of the two biomarkers and self-report at the remaining three (3, 6, and 12 months postquit). The participant's gender, age, marital status, education level, employment status, annual family income, and medical insurance coverage were recorded. Years of US residency were used as a proxy measure of acculturation. We also collected the following information: age at smoking onset, the average number of cigarettes smoked per day, presence of another smoker in the house, indoor-house smoking at home, indoor-office smoking at work, and any past-year quit attempt with which abstinence lasted at least 24 hours.

#### 2.2.1. Fagerström Test for Nicotine Dependence (FTND)

This scale consists of six items assessing the intensity of physiological dependence on nicotine, with four dichotomous items and two 4-point items (0-3) [25]. The scale score can range from 0 to 10. The scale's Cronbach's alpha ranged from 0.55 to 0.74 (e.g., [26–28]). It is believed that the violation of tau equivalence assumed in the estimation of internal reliability is the main reason for low reliability coefficients [29]. Nevertheless, the FTND scale has been used most widely in smoking cessation studies, and a Cronbach's alpha of 0.59 was obtained in the present study.

### 2.3. Data Analysis

Data were analyzed using Stata software (v. 15.0; StataCorp LP, College Station, TX, USA). Baseline participant characteristics were compared between men and women using the Wilcoxon rank-sum test for quantitative data and *χ*^2^ tests for categorical variables. The ROC curve was created by plotting the true positive rate against the false positive rate at various threshold settings to assess diagnostic accuracy [30, 31]. The area under the ROC curve (AUC) ranges from 0 to 1. The area of 1 represents a perfect test; i.e., the screening measure reliably distinguishes between people with positive and negative test results; an area of 0.5 represents a worthless test; i.e., the predictor's discriminating ability is no better than chance [31]. AUC between 0.80 and 0.90 is considered good, whereas an AUC > 0.90 is considered excellent. The optimal cutoff of each measure was determined by comparing the AUC across four CO cutoffs (4-7 ppm) and two cotinine cutoffs (levels 1 and 2) with the “roccomp” command in Stata software.

The ROC curves are a useful way to interpret sensitivity and specificity levels and to determine related cutoffs. Sensitivity was the proportion of all self-reported smokers and participants who yielded an exhaled CO reading at or above a cutoff and a salivary cotinine level at or above a cutoff, and specificity was the proportion of participants who self-reported abstinence and had an exhaled CO reading below a cutoff and a salivary cotinine level below a cutoff. ROC curve analyses not only provide information about cutoff scores but also provide a common natural scale for comparing different predictors (e.g., exhaled CO and salivary cotinine) that are measured in different units. Sensitivity and specificity were estimated, using ROC analyses with the “roctab” command in Stata software.

## 3. Results

Baseline demographics and smoking-related variables were compared between men and women (Table 1). Women were less likely than men to be married (*p* = 0.03) and to have a past-year quit attempt (*p* = 0.02) but were more likely to have medical insurance (*p* = 0.01) and to live with a family member who was also a smoker (*p* < 0.01). Women had their smoking onset at an older age (*p* < 0.01) and smoked fewer cigarettes per day (*p* < 0.001) than men. However, there was no gender difference in nicotine dependence scores and CO readings.

**Figure 1:**
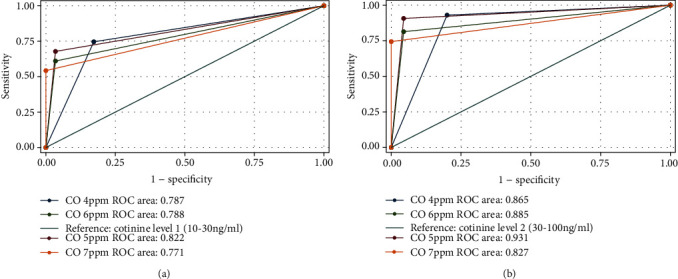
(a) Areas under the ROC curve by four different cutoffs of exhaled CO using salivary cotinine with a cutoff of level 1 (10-30 ng/ml) as the reference at 3 months postquit. (b) Areas under the ROC curve by four different cutoffs of exhaled CO using salivary cotinine with a cutoff of level 2 (30-100 ng/ml) as the reference at 3 months postquit.

The number of participants who were present at each postquit assessment showed almost no change (Table 2). All participants completed an exhaled CO test at each assessment. In contrast, several participants did not complete a salivary cotinine test; two and seven did not do the cotinine test at 6 and 12 months postquit, respectively. They either used a nicotine patch or gum during the past 7 days or refused to do the test. Participants who refused the salivary cotinine test were coded as smokers. ROC curves using the CO readings at postquit assessments were compared across the various CO cutoffs (i.e., 4-7 ppm) against the two cotinine cutoffs between level 1 (10-30 ng/ml, Figure 1(a)) and level 2 (30-100 ng/ml, Figure 1(b)). The AUC had its maximum value for smoking with a CO cutoff of ≥5 ppm and with a cotinine cutoff of ≥level 2. Thus, in this study, true positive smoking was determined by a combination of the CO cutoff of 5 ppm and the cotinine cutoff of level 2. Individuals were classified as smokers if they self-reported smoking, regardless of their CO and cotinine readings.

The lowest agreement (88.6%) between self-reports and biomarkers was observed at 6 months postquit, where 42 participants self-reported abstinence; however, eight of them were classified as smokers using the CO cutoff of ≥5 ppm and the cotinine cutoff of ≥ level 2. Of the eight, five yielded exhaled CO readings as low as 1-2 ppm, but their cotinine readings were level 2 or higher. The highest agreement (97.7%) between self-reports and biomarkers was observed at 12 months postquit. Participants who yielded CO readings below 5 ppm but were classified as smokers were either intermittent or mild smokers (e.g., 2 cigarettes a day). Three participants (one at 3 months and two at 6 months postquit), who yielded cotinine level 1, self-reported smoking 7 to 13 cigarettes a day during the 7 days prior, and their CO readings ranged from 6 to 18 ppm. Interestingly, among those who self-reported smoking, none had both CO and cotinine readings below the cutoffs.

Table 3 shows changes in the sensitivity and specificity of exhaled CO at different cutoffs. The CO cutoff of 5 ppm had the highest sensitivity to detect smokers across all postquit assessments; its specificity ranged 81-98%. The AUC values (boldfaced in Table 3) for exhaled CO were the highest at the cutoff of ≥5 ppm.  Table 4 shows the sensitivity and specificity of the two salivary cotinine cutoffs. The cutoff of ≥level 2 had lower sensitivity but had higher specificity than the cutoff of ≥level 1. The AUC values were all higher with the cutoff of ≥level 2 (boldfaced in Table 4) than with the cutoff of ≥level 1. The AUC values were significantly different between exhaled CO (0.99) and salivary cotinine (0.96, *χ*^2^ = 4.23, *p* = 0.04) at 12 months postquit. The AUC values at 3 and 6 months postquit showed no difference.

## 4. Discussion

To our knowledge, our study is the first of its kind examining the sensitivity and specificity of exhaled CO and salivary cotinine at different cutoffs in Asian Americans. An AUC between 0.80 and 0.90 is considered good, and an AUC > 0.90 is considered excellent. The AUC values for the two measures were all above 90%, i.e., excellent at the CO cutoff of ≥5 ppm and the cotinine cutoff of ≥level 2. The AUC values for exhaled CO significantly improved at 3, 6, and 12 months postquit than those from 1 month postquit at which salivary cotinine tests were not conducted. Interestingly, none of those who self-reported smoking yielded CO and cotinine readings that were both below the cutoffs. These findings suggest that the diagnostic accuracies of the two biomarkers were greatly improved when they were used in combination. Our findings are in support of the recommendation that a combination of biochemical assays may be necessary to biochemically verify abstinence [13].

Although exhaled CO and salivary cotinine generally had similar sensitivity and specificity, CO outperformed cotinine in specificity detecting smokers at 12 months postquit; however, this might be related to the fact that fewer participants performed the cotinine test than the CO test at the time. All individuals who were available at the time performed the CO test, but seven did not do the cotinine test because they either used NRT or refused to do the test. As noted by Benowitz et al. [13], the major limitation of cotinine is that it cannot be used during NRT administration. Compared to the breath CO test, the salivary cotinine test is more cumbersome to perform because individuals need to spit out a large volume of saliva into a collection tube. Furthermore, the test takes a much longer time to yield a result than the CO test (e.g., 30 minutes versus 15 seconds). These factors might have contributed to some participants' refusal of the test.

Similar to our findings, several studies (e.g., [16, 32, 33]) reported that the exhaled CO cutoff of 5 ppm most accurately distinguishes smokers from nonsmokers. However, Cropsey et al. [22] reported the cutoff of 3 ppm having the best efficiency in detecting true smokers and false-negative smokers. MacLaren et al. [32] used the Bedfont monitor, whereas the remaining three [16, 22, 33] used the Vitalograph monitor. Different CO readings have been reported between the two monitors. For example, Karelitz et al. [34] reported that the Bedfont monitor (South Hackensack, NJ, USA) gave mean CO readings 3.8 ppm higher among regular smokers and 1.7 ppm higher among those who reported 12-24-hour abstinence than the Vitalograph monitor (Lenexa, KS, USA). Because of this notable difference, Benowitz et al. [13] recommended in the 2019 update for biochemical verification of tobacco use and abstinence that researchers report details of analytic methodology, including the type of a CO monitor used.

Of note, three participants in our study who reported smoking 7-13 cigarettes per day yielded cotinine level 1. Similar findings were reported by Marrone et al. [16]. In their study, 23.3% of smokers (12 of 46 heavy and nine of 44 light smokers) had saliva NicAlert® readings in level 0 (0–10 ng/ml cotinine). In contrast, Etter [15] reported that among 82 self-reported nonsmokers who yielded NicAlert® readings of level 1 (10-30 ng/ml), only two (2.4%) had values within the purported range, and 71 (86.6%) were below 4 ng/ml when compared with the readings of liquid chromatography-tandem mass spectrometry that is often used as the gold standard. Anderson et al. [35] postulated that certain drugs and dietary substances containing a pyridine ring could interfere with the assay by overestimating cotinine concentration. Because of this lack of specificity, it is often reported that NicAlert® test kits are more likely to give false positives than false negatives [13, 15]. Given these conflicting findings, salivary NicAlert® test kits can be best used as a secondary measure to assist other indicators such as exhaled CO and urinary cotinine.

The major limitation of this study was that we used a NicAlert® test kit that yields a crude semiquantitative estimate of cotinine. Another limitation was the relatively smaller number of women in our sample. We were not able to compare gender differences in the cutoffs of the two biomarkers. Given some notable differences in demographics and smoking-related variables found between Korean American men and women, future studies should oversample Korean women and explore any possible gender differences in the sensitivity and specificity of the two biomarkers while comparing AUC values with different cutoffs. Despite the limitations stated above, our study was able to determine the optimal cutoffs of exhaled CO and salivary cotinine biomarkers for Korean Americans. Our findings are generally in support of the recommendations made by Benowitz et al. [13].

In 2018, fewer than one out of ten (7.5%) adult smokers in the United States were able to succeed in quitting during the past year [21]. Clinicians and researchers should collaborate to develop effective intervention strategies to help smokers attain complete abstinence. While doing so, they must include a plan to verify abstinence using biochemical measures with optimal cutoffs. As our study findings suggest, a combination of exhaled CO and salivary cotinine seems to be an ideal approach to the verification of self-reported abstinence among individuals who are likely to underreport the use during and after treatment.

## Figures and Tables

**Table 1:** Baseline demographics and smoking behavior variables (*N* = 88).

Variable (range)	Male (*n* = 76)	Female (*n* = 12)	*p* value^a^
	Mean ± SD/*n* (%)	Mean ± SD/*n* (%)	
Age (28~72)	49.8 ± 9.1	48.8 ± 9.8	ns
Marital status			0.028
Married	66 (86.8%)	7 (58.3%)	
All others	10 (13.2%)	5 (41.7%)	
Education level			ns
≤High school	19 (25.0%)	3 (25.0%)	
Some years in college	9 (11.8%)	2 (16.7%)	
4-year college degree	45 (59.2%)	5 (41.6%)	
Graduate degree	3 (4.0%)	2 (16.7%)	
Employment status			ns
Full-time employed	70 (92.1%)	9 (75.0%)	
All others	6 (7.9%)	3 (25.0%)	
Annual family income			ns
<$20,000	9 (11.9%)	1 (8.3%)	
$20,000~$39,999	15 (19.7%)	2 (16.7%)	
$40,000~$79,999	36 (47.4%)	8 (66.7%)	
$80,000-$99,999	8 (10.5%)	0 (0.0%)	
$100,000~	8 (10.5%)	1 (8.3%)	
Availability of medical insurance			0.013
Yes	27 (35.5%)	9 (75.0%)	
No	49 (64.5%)	3 (25.0%)	
Years in the US (1~37)	17.7 ± 8.4	20.8 ± 10.7	ns
Age at smoking onset (14~38)	19.1 ± 2.7	23.6 ± 6.1	0.008
Average number of cigarettes smoked per day (10~35)	17.7 ± 5.8	11.7 ± 2.1	0.0004
Nicotine dependence (0~9)	4.8 ± 2.1	4.8 ± 1.7	ns
Other smokers in the household			0.003
Yes	16 (21.1%)	8 (66.7%)	
No	60 (78.9%)	4 (33.3%)	
Smoking in indoor house			ns
Yes	22 (28.9%)	6 (50.0%)	
No	54 (71.1%)	6 (50.0%)	
Smoking in indoor offices at work			ns
Yes	32 (42.1%)	3 (25.0%)	
No	44 (57.9%)	9 (75.0%)	
Any 24-hour abstinence in the past year			0.024
Yes	48 (63.2%)	3 (25.0%)	
No	28 (36.8%)	9 (75.0%)	
Baseline carbon monoxide level (6~59)	23.8 ± 11.4	20.7 ± 9.9	ns

^a^ *p* values by the Wilcoxon rank-sum or *χ*^2^ test. SD = standard deviation; *n* = number; ns = not significant.

**Table 2:** The number of participants completed each follow-up assessment and CO and cotinine measures.

Month at follow-up	1 month	3 months	6 months	12 months
Follow-up data completed	82	80	78	79
Breath CO test done	82	80	78	79
Salivary cotinine test done	na^a^	80	76	72
Self-reported abstinence	69	50	42	32
Abstinence verified with biochemical measures^b^	62	43	34	32

^a^Not applicable. ^b^Determined by exhaled CO ≤ 4 ppm and salivary cotinine ≤ level 1 (10-30 ng/ml) except for a 1-month follow-up at which abstinence was determined by exhaled CO only.

**Table 3:** Sensitivity and specificity of various breath carbon monoxide cutoff levels at each follow-up.

CO cutoff (ppm)	Follow-up	Sensitivity	Specificity	ROC area
4	1 month	0.923	0.726	0.824
	3 months	0.933	0.837	0.885
	6 months	0.889	0.912	0.900
	12 months	0.983	0.710	0.846
5	1 month	0.808	1.000	**0.904**
	3 months	0.911	1.000	**0.956**
	6 months	0.889	1.000	**0.944**
	12 months	0.983	1.000	**0.991**
6	1 month	0.654	1.000	0.827
	3 months	0.822	1.000	0.911
	6 months	0.870	1.000	0.935
	12 months	0.930	1.000	0.965
7	1 month	0.500	1.000	0.750
	3 months	0.711	1.000	0.856
	6 months	0.852	1.000	0.926
	12 months	0.930	1.000	0.965

The number of participants varied at each follow-up: 82 at 1 month, 80 at 3 months, 78 at 6 months, and 79 at 12 months.

**Table 4:** Sensitivity and specificity of salivary cotinine cutoff levels 1 and 2 at each follow-up.

Cotinine cutoff (ng/ml)	Follow-up	Sensitivity	Specificity	ROC area
Level 1: 10-30	3-M	0.978	0.651	0.815
	6-M	0.982	0.647	0.814
	12-M	1.000	0.844	0.922
Level 2: 30-100	3-M	0.956	1.000	**0.978**
	6-M	0.944	1.000	**0.972**
	12-M	0.912	1.000	**0.956**

The number of participants varied at each follow-up: 80 at 3 months, 76 at 6 months, and 72 at 12 months.

## Data Availability

The data used in this study are available upon request by contacting the corresponding author Dr. Sun S. Kim at sun.kim@umb.edu.
